# Rates of Low-Value Service in Australian Public Hospitals and the Association With Patient Insurance Status

**DOI:** 10.1001/jamanetworkopen.2021.38543

**Published:** 2021-12-10

**Authors:** Juliana de Oliveira Costa, Sallie-Anne Pearson, Adam G. Elshaug, Kees van Gool, Louisa R. Jorm, Michael O. Falster

**Affiliations:** 1 Centre for Big Data Research in Health, Faculty of Medicine, UNSW Sydney, Kensington, New South Wales, Australia; 2 Centre for Health Policy, Melbourne School of Population and Global Health, The University of Melbourne, Melbourne, Victoria, Australia; 3 Centre for Health Economics Research and Evaluation, University of Technology Sydney, Sydney, New South Wales, Australia

## Abstract

**Importance:**

Low-value services have limited or no benefit to patients. Rates of low-value service in public hospitals may vary by patient insurance status, given that there may be different financial incentives for treatment of privately insured patients.

**Objective:**

To assess the variation in rates of 5 low-value services performed in Australian public hospitals according to patient funding status (ie, private or public).

**Design, Setting, and Participants:**

This retrospective cross-sectional study analyzed New South Wales public hospital data from January 2013 to June 2018. Patients included in the sample were over age 18 years and eligible to receive low-value services based on diagnoses and concomitant procedures. Data analysis was conducted from June to December 2020.

**Main Outcomes and Measures:**

Hospital-specific rates of low-value knee arthroscopic debridement, vertebroplasty for osteoporotic spinal fractures, hyperbaric oxygen therapy, oophorectomy with hysterectomy, and laparoscopic uterine nerve ablation for chronic pelvic pain were measured. For each measure, rates within each public hospital were compared by patient funding status descriptively and using multilevel models.

**Results:**

A total of 219 862 inpatients were included in analysis from 58 public hospitals across the 5 measures. A total of 38 365 (22 904 [59.7%] women; 12 448 [32.4%] aged 71-80 years) were eligible for knee arthroscopic debridement for osteoarthritis; 2520 (1924 [76.3%] women; 662 [26.3%] aged 71-80 years), vertebroplasty for osteoporotic spinal fractures; 162 285 (82 046 [50.6%] women; 28 255 [17.4%] aged 61-70 years), hyperbaric oxygen therapy; 15 916 (7126 [44.8%] aged 41-50 years), oophorectomy with hysterectomy; and 776 (327 [42.1%] aged 18-30 years), uterine nerve ablation for chronic pelvic pain. Overall rates of low-value services varied considerably between measures, with the lowest rate for hyperbaric oxygen therapy (0.3 procedures per 1000 inpatients [47 of 158 220 eligible inpatients]) and the highest for vertebroplasty (30.8 procedures per 1000 eligible patients [77 of 2501 eligible inpatients]). There was significant variation in rates between hospitals, with a few outlying hospitals (ie, <10), particularly for knee arthroscopy (range from 1.8 to 21.0 per 1000 eligible patients) and vertebroplasty (range from 13.1 to 70.4 per 1000 eligible patients), with higher numerical rates of low-value services among patients with private insurance than for those without. However, there was no association overall between patient insurance status and low-value services. Overall differences in rates among those with and without private insurance by individual procedure type were not statistically significant.

**Conclusions and Relevance:**

There was significant variation in rates of low-value services in public hospitals. While there was no overall association between private insurance and rate of low-value services, private insurance may be associated with low-value service rates in some hospitals. Further exploration of factors specific to local hospitals and practices are needed to reduce this unnecessary care.

## Key Points

**Question:**

Do inpatients with supplemental private insurance coverage have higher rates of low-value services than those without private insurance in Australian public hospitals?

**Findings:**

In this cross-sectional study of 219 862 inpatients, large variations in rates of low-value services were observed across hospitals. Overall, private insurance was not associated with the receipt of low-value services; however, a small number of outlying hospitals had higher rates of low-value services among privately insured patients.

**Meaning:**

These results suggest that reducing low-value services requires investigating these practices at the hospital level.

## Teaser

This cross-sectional study compares rates at which low-value services were used for adult inpatients with private vs public insurance in public hospitals in New South Wales, Australia.

## Introduction

Hospitals account for the highest proportion of health spending worldwide; optimal resource allocation in this setting is key to improving health system efficiency and sustainability. Some tests, procedures, and services are considered to be low-value care, as they are not beneficial to patients or their risks outweigh expected benefits.^1^ Identifying and curtailing low-value care will curb unnecessary spending and improve quality and outcomes of care.^1,2^ Rates of low-value care have been found to vary between treating facilities^3,4^ and are associated with patient characteristics (eg, gender, age), access to services (eg, availability and frequency of specialist consultations^5^), and physician treatment preferences.^6^ Identifying health system factors influencing the provision of low-value care can provide opportunities to reduce this unnecessary care.

One factor potentially influencing low-value care is private health insurance. In the US, commercial insurers negotiate reimbursement for procedures directly with hospitals and doctors, leading to large variation in reimbursements and generally higher profit margins for treating commercially insured patients rather than Medicare patients.^5,7^ These financial incentives may motivate the provision of low-value care in insured populations, although the findings from studies exploring this association are inconsistent.^5,7,8,9^ While treatment decisions are likely influenced by patient and physician preferences, previous studies have not investigated differences or accounted for differences in hospital-level care—where such disparities are likely to occur.

Few studies have investigated associations between health insurance and low-value care outside the US. Australia has a mixed public and private health system that offers unique insights on the role of private health insurance in a system providing universal health care.^10,11^ Approximately 46% of the population hold private health insurance, which, unlike the US, complements the public system through access to private hospitals (primarily elective procedures) and some outpatient services. Patients with private health insurance retain the right to be admitted to public hospitals as a public patient, but they also have the option to be admitted as a private patient. Should they elect to be treated as a private patient, they receive some benefits—such as choice of treating physician for elective surgery (including potential shorter waiting times^12^) and a private room (if available)—although they may face additional out-of-pocket costs. As with the US, there are incentives for physicians and public hospitals to treat patients with private insurance, including cost-shifting to private health insurers and additional funding from patient out-of-pocket costs (by billing procedures above the standard rebate).^10,13^ Potential incentives through activity-based funding are also available,^14^ whereby hospitals receive multiple reimbursements for select patient costs (eg, procedures, prostheses, and accommodation) from different funding agents (eg, state or federal government, health insurers) depending on the funding arrangements in place.^15^ For many Australian public hospitals, there are funding implications for meeting local targets to treat private patients,^14^ and it is likely that patients who choose to use private health insurance in public hospitals are influenced by jurisdictional policies and incentives.^16^

The rapid increase in the proportion of privately funded hospitalizations within Australian public hospitals has raised concerns that patients without private insurance will experience delays in access to care, as well as concerns over the increased financial burden to health insurers.^14^ While we know there are differences in types of care received in public and private health systems,^17^ little is known about whether private funding influences care received within public hospitals.^3,18^ In this study, we explored the variation in rates of 5 low-value services within New South Wales (NSW) public hospitals and assessed whether rates of those services differed between the public vs private status of inpatients within the same hospital.

## Methods

### Data Set and Study Population

We conducted a population-based cross-sectional study using deidentified data from the NSW Admitted Patient Data Collection, a census of all inpatient discharges, transfers, and deaths from NSW public and private hospitals. The census contains data relating to patient demographics (eg, age, sex, and area of residence), admission characteristics (eg, private health insurance status; diagnoses, coded according to the *International Classification of Diseases 10th Revision Australian Modification* [*ICD-10-AM*]; and procedures, coded according to the Australian Classification of Health Interventions [ACHI]). NSW is the most populous Australian state (7.9 million in 2017) and has 221 public and 210 private hospitals.^19^

We included adult patients (aged ≥18 years) admitted to NSW public hospitals between January 2013 and June 2018. Patients with a funding status other than public or private were excluded (ie, veterans, workers compensation). This research was approved by the NSW Population and Health Services Research Ethics Committee with a waiver of informed consent. We followed the Strengthening the Reporting of Observational Studies in Epidemiology (STROBE) reporting guideline for observational studies.

### Outcomes

We measured 5 low-value services in NSW public hospitals using existing indicators developed for use in Australia: knee arthroscopic debridement, vertebroplasty for osteoporotic spinal fractures, hyperbaric oxygen therapy for various indications, oophorectomy with hysterectomy, and laparoscopic uterine nerve ablation for chronic pelvic pain.^4,20^ These procedures are recognized as do-not-do treatments for specific patient populations owing to the lack of evidence of their effectiveness or robust evidence of their ineffectiveness.^20^ The measures are considered conservative lower-bound estimates of the low-value care procedure, given they have strict criteria identifying patients for whom treatment is inappropriate.

We measured each low-value service separately. For each service, we identified eligible patients who could potentially receive the service but for whom it is not indicated and considered low value based on demographics, diagnoses, and procedures recorded in the admission (eTable 1 in the Supplement).^4,20^ We then identified the provision of low-value services among these patients. An example of a low-value service by these criteria would be oophorectomy during hysterectomy (after excluding patients with gynecological cancers or endometriosis, where the procedure is justifiable). Such a patient-indication approach to measuring low-value services can be used to identify differences in treatment patterns between patient groups admitted for specific types of conditions.^2^

Not all hospitals provide the services evaluated, so we excluded hospitals where fewer than 5 patients received the procedure (low-value or otherwise) in the period assessed (eTable 2 in the Supplement).^20^ Where patients had changes to the type of care within a hospital (eg, from acute to subacute care) or transfers between hospitals, these were treated as a single hospital stay at the hospital of admission.

### Statistical Analysis

#### Rates of Low-Value Services

We quantified the occurrence of low-value services for each measure according to patient funding status (ie, private or public). For patients with multiple episodes of care within a hospital stay, we considered them to be a patient with private insurance if any episodes were billed using private health insurance.

As patients could have multiple hospital admissions but would not be expected to receive the service on all admissions (eg, rehabilitation admissions for osteoarthritis), we calculated rates of low-value services on a per-patient basis (ie, rates of each low-value service per 1000 eligible patients). We counted the number of eligible patients and low-value services according to each funding category, and where patients had multiple hospitals stays in different funding categories, the patient contributed to each category accordingly.

#### Variation Between Public Hospitals in Rates of Low-Value Services

We projected rates of low-value services within each hospital, with the assumption that the age (10-year age groups) and sex distribution of patients in each hospital was the same as for the entire cohort of eligible patients for each measure. We estimated this rate using multilevel logistic models, with patients clustered in their hospital of admission as a random intercept. We tested for significance of the random effect using a Wald test. We quantified between-hospital variation using the random intercept parameter for the hospital of admission. The estimated rate of each low-value service was calculated for each hospital from the combination of patient-level effects (parameter estimates for average patient characteristics) and the hospital-level random effect, and then graphically explored using caterpillar plots. We did not assess any hospital variation for laparoscopic uterine nerve ablation because of the limited data available.

#### Difference in Low-Value Service Rates Between Patients With and Without Private Insurance

We tested for differences in rates of each low-value service between patients with and without private insurance in the same hospital by adding a term for funding status to the multilevel models. As the proportion of patients with private insurance for many hospitals will be affected by the socioeconomic status of the patient population, we further adjusted for socioeconomic disadvantage quintiles and remoteness of the residence.

We assessed the differences in rates of low-value services between publicly and privately insured patients in the same hospital in 2 ways. First, we descriptively compared rates of low-value services between public and private patients within each hospital, presenting comparisons using scatter plots. We statistically tested whether patient insurance status was associated with rates low-value services and the difference between hospitals using random slope multilevel models,^21,22^ adjusting for age, sex, socioeconomic disadvantage, and remoteness of residence, and allowed both the baseline rate of low-value services (ie, random intercept) and the correlation with insurance type (random slope) to vary for each hospital. We used Wald tests to test the significance of the random slopes, and plotted hospital-specific effects as odds ratios (ORs) by combining the fixed effect of insurance type with the residual random effect.

We performed statistical analyses using SAS version 9.4 (SAS Institute Inc), and multilevel modeling in MLwiN version 3.05 (Centre for Multilevel Modeling) using Markov chain Monte Carlo estimation for random slope models. We produced graphics using R version 4.1.0 (R Core Team 2021). For all statistical analyses, we considered the level of significance *P* < .05 using 2-sided tests.

## Results

We identified public hospitals where 5 or more admitted patients received each service of interest (eTable 2 in the Supplement). This ranged from 3 hospitals for uterine nerve ablation to more than 50 hospitals for oophorectomy or knee arthroscopic debridement. Within these hospitals, we identified 219 862 patients eligible to receive at least 1 of the services, ranging from fewer than 800 patients for laparoscopic uterine nerve ablation to 158 220 patients for hyperbaric oxygen therapy (Table 1). As expected, the demographic composition of patients with and without private insurance and the number of eligible patients differed within each measure (eTable 3 in the Supplement). A total of 38 365 (22 904 [59.7%] women; 12 448 [32.4%] aged 71-80 years) were eligible for knee arthroscopic debridement for osteoarthritis; 2520 (1924 [76.3%] women; 662 [26.3%] aged 71-80 years), vertebroplasty for osteoporotic spinal fractures; 162 285 (82 046 [50.6%] women; 28 255 [17.4%] aged 61-70 years); hyperbaric oxygen therapy; 15 916 (7126 [44.8%] aged 41-50 years), oophorectomy with hysterectomy; and 776 (327 [42.1%] aged 18-30 years), uterine nerve ablation for chronic pelvic pain. Across all measures, patients with private insurance tended to be slightly older than patients with public insurance, and a larger proportion lived in major cities and inner regional areas (ie, geographic areas with few restrictions on access to goods and services).

**Table 1.** Low-Value Services Among Patients Admitted to Public Hospitals by Patient Funding Status^a^

	No. of eligible patients^b^	No. of low-value services	Rate per 1000 inpatients
Knee arthroscopic debridement for osteoarthritis			
Overall	38 188	264	6.9
Public inpatients	34 329	254	7.4
Private inpatients	4036	10	2.5
Vertebroplasty for osteoporotic spinal fractures			
Overall	2501	77	30.8
Public inpatients	1591	46	28.9
Private inpatients	929	32	34.4
Hyperbaric oxygen therapy for various indications			
Overall	158 220	47	0.3
Public inpatients	119 044	33	0.3
Private inpatients	43 241	16	0.4
Oophorectomy with hysterectomy			
Overall	15 915	66	4.1
Public inpatients	14 354	56	3.9
Private inpatients	1569	10	6.4
Laparoscopic uterine nerve ablation for chronic pelvic pain^c^			
Overall	770-780	<10	11.6
Public inpatients	690-700	<10	11.5
Private inpatients	80-90	<10	12.2

^a^ Services to patients with multiple admissions under public and private insurance were considered in each group as appropriate; overall numbers may differ from the sum of public and private accordingly.

^b^ Eligible patients are those who could potentially receive the service, but for whom it is considered to be low value.

^c^ For privacy reasons, we used consequential cell suppression for small numbers (<10). Counts have been presented as a range accordingly.

### Rates of Low-Value Services in Public Hospitals

Overall rates of low-value services varied across the 5 services, with the highest rates for vertebroplasty for osteoporotic spinal fractures (30.8 per 1000 [77 of 2501] eligible patients) and laparoscopic uterine nerve ablation for chronic pelvic pain (11.6 per 1000 [<10 of 770-780] eligible patients), and the lowest for hyperbaric oxygen therapy (0.3 per 1000 [47 of 158 220] eligible patients) (Table 1).

### Variation Between Public Hospitals in Rates of Low-Value Services

**Figure 1.**
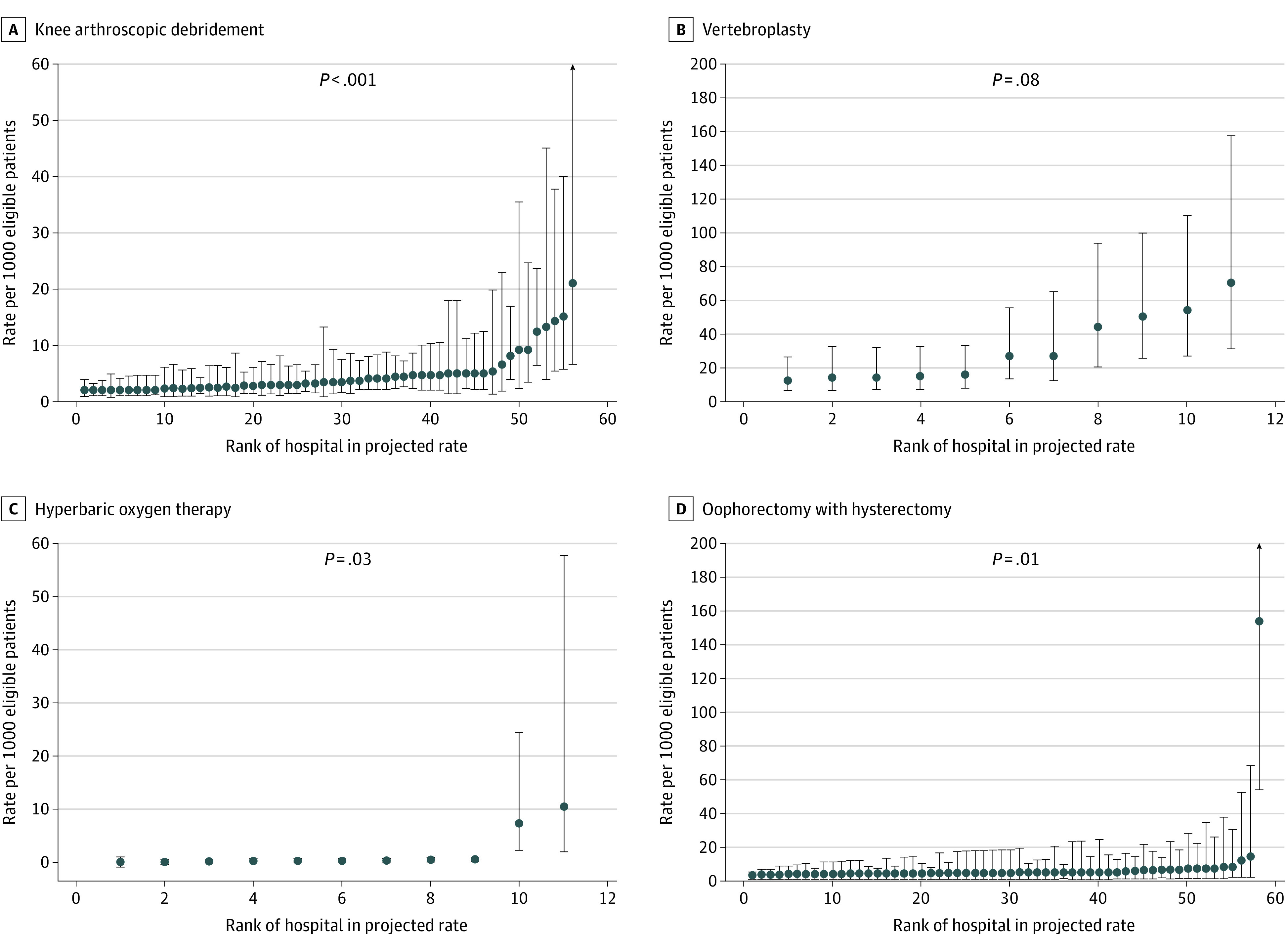
Projected Rates of Low-Value Services in Public Hospitals Assuming Similar Distribution of Age and Sex Within Each Hospital Public hospitals with at least 5 admitted patients receiving the service between January 2013 to June 2018, irrespective if the service is considered low value or not. *P* values from Wald test of random intercept on the hospital of admission.

We observed substantial variation in projected rates of low-value services across hospitals (Figure 1), particularly for knee arthroscopy (range from 1.8 to 21.0 per 1000 eligible patients) and vertebroplasty (range from 13.1 to 70.4 per 1000 eligible patients). Most hospitals had similar rates within each measure of low-value services, although there were 1 or 2 outlying hospitals with very high rates of low-value oophorectomy and hyperbaric oxygen therapy.

### Difference in Rates of Low-Value Services Between Patients With and Without Private Insurance

Patients with private insurance did not have significantly higher rates of low-value services than patients without for 4 of the 5 measures (Table 2). For knee arthroscopic debridement, the point estimate was lower for patients with private insurance compared with those without, but the result was not significant (aOR, 0.57; 95% CI, 0.32-1.02). Similar patterns were observed when further adjusting for socioeconomic status and geographic remoteness of area of residence (Table 2).

**Table 2.** Difference in Rates of Low-Value Services Between Inpatients With and Without Private Insurance Within Public Hospitals

Measure	aOR (95% CI)^a^	
	Age and sex	Age, sex, and the remoteness and SES of area^b^
Knee arthroscopic debridement for osteoarthritis	0.57 (0.32-1.02)	0.56 (0.31-1.01)
Vertebroplasty for osteoporotic spinal fractures	1.09 (0.68-1.75)	1.08 (0.66-1.76)
Hyperbaric oxygen therapy for various indications	1.31 (0.78-2.19)	1.53 (0.92-2.54)
Oophorectomy with hysterectomy^c^	1.78 (0.87-3.63)	1.77 (0.88-3.54)

Abbreviations: aOR, adjusted odds ratio; SES, socioeconomic status.

^a^ ORs higher than 1 indicate a higher rate of low-value services among inpatients with private insurance.

^b^ Remoteness and SES according to geographic area (statistical area 2) of residence. Remoteness categorized by Australian Bureau of Statistics Remoteness Areas; SES categorized by Australian Bureau of Statistics Socio-Economic Indexes for Areas Index of Relative Socio-Economic Disadvantage.

^c^ Age adjusted only, as measure is restricted to women only.

**Figure 2.**
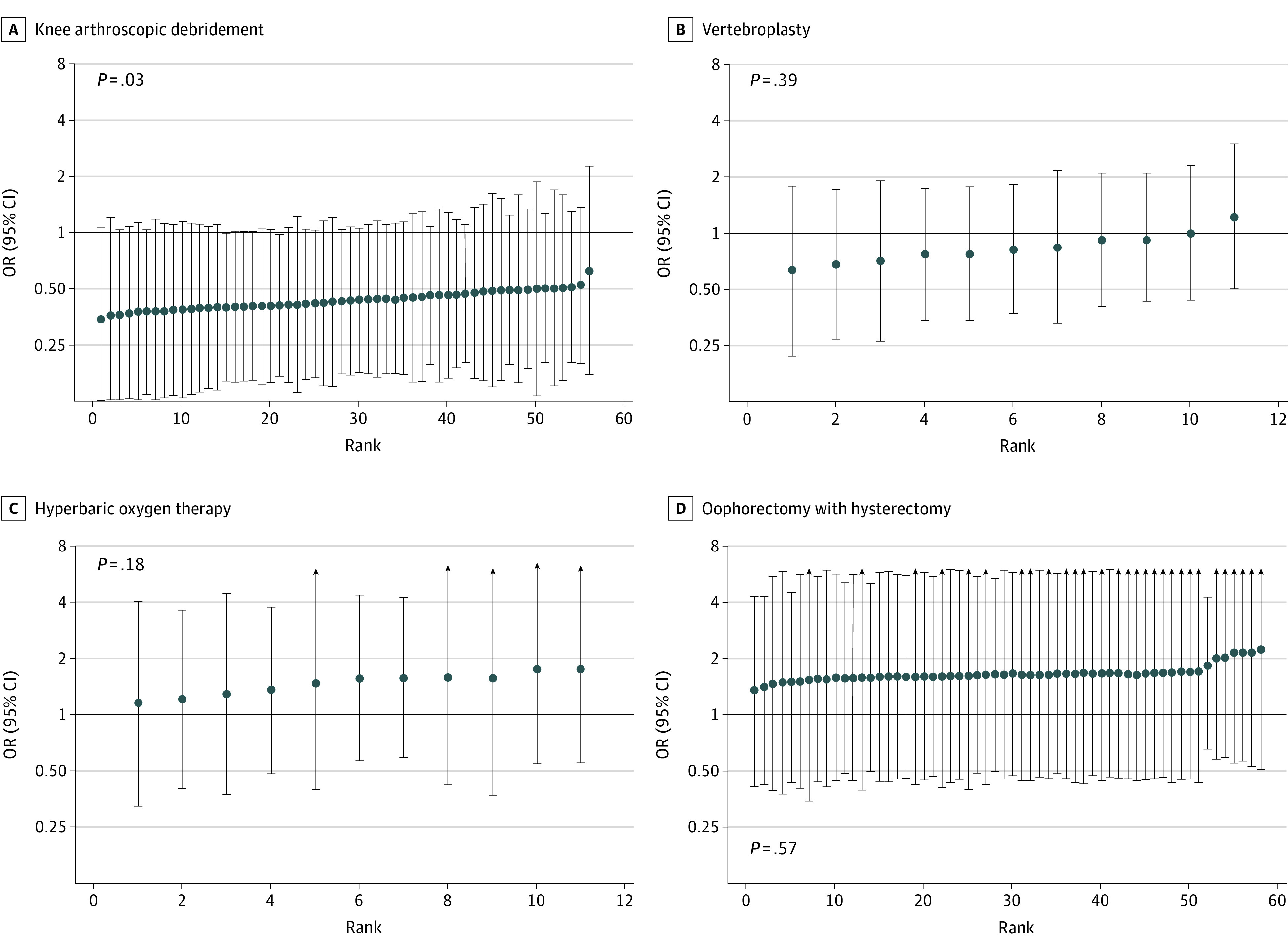
Difference in Rates of Low-Value Services Between Private and Public Inpatients Within New South Wales Public Hospitals Odds ratios (ORs) higher than 1 indicate a higher rate of low-value services among inpatients with private insurance. ORs from the random slope multilevel logistic model, adjusted for age (10-year age groups), sex, remoteness, and socio-economic disadvantage of area of residence. Public hospitals with at least 5 admitted patients receiving the service between January 2013 to June 2018, irrespective of whether the service is considered low-value or not. *P* values from Wald test for random slope testing between-hospital variation in the association between private health insurance with rates of low-value care.

When comparing how rates of low-value services varied between patients with and without private insurance within the same hospital, we did not find a consistent pattern in the descriptive (eFigure in the Supplement) or adjusted analyses (Figure 2). Hospitals had either higher or lower rates of low-value services among patients with private insurance, with most hospitals having low numbers of the low-value services measured. However, there were some clearly outlying hospitals (<10) with higher rates of low-value services among patients with private insurance (eFigure in the Supplement).

After adjustment, we did not find significant differences between hospitals in the association of private health insurance with rates of low-value services (Figure 2). While there was some indication of a direction of the outcome in each hospital (eg, private patients had consistently lower rates of knee arthroscopy, consistently higher rates of hyperbaric oxygen therapy and oophorectomy, and heterogenous findings for vertebroplasty), there was high uncertainty in estimates because of low number of events, as represented by the large confidence intervals.

## Discussion

We found rates of low-value services differed across NSW public hospitals. However, we identified potentially higher rates of low-value care among privately insured patients in a small number of hospitals. Our findings highlight the need to explore local patterns of care when investigating factors associated with low-value care.

International studies have similarly found inconsistent associations between patient funding and rates of low-value services.^3,5,7,8,23,24^ Variations in low-value services are attributed mostly to treating hospital and clinicians than patients.^3,25^ As there are multiple pathways through which insurance may affect service provision (such as the need for prior examinations, referrals, or preauthorization for different procedures),^7,26^ it is likely that the health system factors driving low-value services will differ according to local health system characteristics and the services measured. For example, US and Swiss studies found higher rates in privately insured patients,^7,27^ while we observed lower rates of low-value knee arthroscopy performed in public hospitals among private inpatients compared with public inpatients with osteoarthritis. However, public hospitals in Australia may have different admission policies and practices in treating privately insured patients with osteoarthritis of the knee (eg, patients more often admitted for rehabilitation), which alongside the complementary role of private hospitals, may factor into the rates at which low-value services are offered through differences in the types of eligible patients admitted.^28^

We did not observe significantly higher rates of low-value services for patients with private insurance compared with those without who were admitted to public hospitals for the conditions of interest. This finding suggests that potential funding inequities driving increased admission of privately insured patients in public hospitals (eg, through out-of-pocket costs and additional reimbursement of procedures, prostheses, and accommodation)^14^ are not necessarily incentivizing wasteful and inefficient care. Addressing unwarranted clinical variation involves identifying local variations in practice and the treatment decisions that affect patterns of care. To our knowledge, this is one of the only studies to explore variation in low-value services at the hospital level. We identified a handful of public hospitals that potentially may be providing low-value services more often to privately insured patients. Reasons for these discrepancies are not fully understood, but may include the financial incentives for some hospitals and doctors to treat these patients,^14,18,25,29^ patient incentives to use their insurance (eg, perceived quality of care, reduced waiting times, increase choice of providers), and differences in treatment for patients based on their insurance status in public and private hospitals.^14,26^

Incentives promoting low-value care extend beyond the public hospital system as well as further studies exploring the broader role of the private sector are required. Many procedures are sensitive to the preferences of physicians and patients, such as the cost and relative availability of alternative treatments, and there may be greater capacity for discretionary care within the private sector and for other types of low-value procedures.^6,27^ Furthermore, the majority of elective procedures occur within the private sector. For example, we found the rate of low-value knee arthroscopic debridement was higher in patients without private insurance in public hospitals, although the majority of overall knee arthroscopic procedures (including low-value procedures) are performed in private hospitals.^24,29^ Similarly, the vast majority of vertebroplasty procedures are privately funded,^30^ with private hospitals in NSW and Western Australia accounting for as much as 63% of all low-value vertebroplasties performed nationally.^20^ There have been efforts to disincentivize some low-value services, and restrictions on government subsidies through the Medicare Benefits Schedule Review Taskforce appear to be reducing the rates of these services.^31,32^ It is important to continue exploring financial and service-related factors that may incentivize a variety of low-value services within both public and private systems, over time and across jurisdictions.

### Limitations

This study had several limitations. We used administrative data to identify and quantify low-value services, which are limited in precision for identifying clinical diagnoses and indications for procedures and do not document the rationale underpinning clinical treatment decisions. However, we used measures developed for Australian data and validated by clinicians to provide the greatest accuracy in identifying do-not-do treatments.^20^ These measures are likely to underestimate the actual number of low-value services provided given our conservative definition and reliance on accurate and complete coding of diagnoses and procedures. The benefit of using conservative measures is greater certainty the care provided is inappropriate. While a broader set of measures of low-value care have been developed for the Australian setting,^33^ we only used those in which codes were available in the public domain.

Our event numbers were low, limiting statistical power and the validity of statistical tests. This may have been owing to the conservative nature of our measures, but may also reflect increased public awareness and the incidence of some measures decreasing accordingly.^4,23,32^ While multilevel models account for uncertainty from low numbers in each hospital through use of a shrinkage factor,^22^ the low prevalence of procedures still limited our analysis options.

Our findings may not be generalizable to other health systems with different funding arrangements for hospital care.^14^ However, many health systems have a mixture of public and private funding, and the Australian experience can inform health systems, such as the US, where expansion of universal health care is being considered.^10^ Further research exploring local treatment decisions for providing low-value care within different health systems, and for a variety of measures of wasteful and inefficient care, are needed.

## Conclusions

There is considerable variation in rates of low-value services across public hospitals. While rates of these services did not differ significantly or consistently according to patients' funding status within the same hospital, private health insurance may be associated with low-value services within some hospitals. Further exploration of the practices that promote low-value care at the local level, as well as within the broader private sector, are needed to reduce this unnecessary care.

## Notes

Supplement. **eTable 1.** Low-Value Services and Eligibility Criteria Used in This Study **eTable 2.** Number of Public Hospitals Performing Services Measured, and the Number of Low-Value Services Provided Within These Hospitals **eTable 3.** Characteristics of Eligible Inpatients According to Funding Status for Each Measure of Low-Value Service **eFigure.** Crude Rates of Low-Value Services (per 1000 Eligible Patients) in Private Compared With Public Inpatients Within Each Public Hospital
