# Supplementary material for: Rates of Low-Value Service in Australian Public Hospitals and the Association With Patient Insurance Status
Source: JAMA Netw Open. 2021 Dec 10;4(12):e2138543. doi: 10.1001/jamanetworkopen.2021.38543 (PMC8665371; doi:10.1001/jamanetworkopen.2021.38543)
Supplement: Supplement. — eTable 1. Low-Value Services and Eligibility Criteria Used in This Study eTable 2. Number of Public Hospitals Performing Services Measured, and the Number of Low-Value Services Provided Within These Hospitals eTable 3. Characteristics of Eligible Inpatients According to Funding Status for Each Measure of Low-Value Service eFigure. Crude Rates of Low-Value Services (per 1000 Eligible Patients) in Private Compared With Public Inpatients Within Each Public Hospital [file jamanetwopen-e2138543-s001.pdf]

Supplemental Online Content

de Oliveira Costa J, Pearson SA, Elshaug AG, van Gool K, Jorm LR, Falster MO. Rates of low-value service in Australian public hospitals and the association with patient insurance status. *JAMA Netw Open.* 2021;4(12):e2138543. doi:10.1001/jamanetworkopen.2021.38543

eTable 1. Low-Value Services and Eligibility Criteria Used in This Study

eTable 2. Number of Public Hospitals Performing Services Measured, and the Number of Low-Value Services Provided Within These Hospitals

eTable 3. Characteristics of Eligible Inpatients According to Funding Status for Each Measure of Low-Value Service

eFigure. Crude Rates of Low-Value Services (per 1000 Eligible Patients) in Private Compared With Public Inpatients Within Each Public Hospital

This supplemental material has been provided by the authors to give readers additional information about their work.

© 2021 de Oliveira Costa J et al. *JAMA Network Open.*

eTable 1. Low-Value Services and Eligibility Criteria Used in this Study (Source: Duckett SJ, Breadon P, Romanes D. Identifying and acting on potentially inappropriate care. The Medical Journal of Australia. 2015; 203: 183e 1-6)

Low-value service	ACHI code procedure	'Eligible' people (based on ICD-10-AM codes and ACHI code procedures)
Arthroscopic debridement for osteoarthritis of the knee	4955800	**Inclusions:**<br>Osteoarthritis: M170, M171, M172, M173, M174, M175, M179<br>**Exclusions:**<br>Meniscal tears: M1126, M224, M23, M230, M2303, M2304, M2306, M2309, M231, M2313, M2316, M232, M2320, M2321, M2322, M2323, M2324, M2325, M2326, M2329, M233, M2330, M2332, M2333, M2334, M2335, M2336, M2339, M2340, M234, M2341, M2342, M2343, M2344, M2345, M2346, M2347, M2349, M2350, M2351, M2352, M2353, M2359, M238, M2380, M2381, M2382, M2383, M2384, M2385, M2386, M2387, M2389, M239, M2391, M2392, M2393, M2394, M2396, M2399, M6596, M6786, M9486, S832, S833, 4950002, 4955702, 4955801, 4956000, 4956003, 4956101, 4956201, 4956300, 4956600, 5012401
Vertebroplasty for osteoporotic spinal fractures	3540000, 3540001	**Inclusions:**<br>Osteoporotic fractures: M8008, M8028, M8058, M8088, M8098
Removal of healthy ovaries during hysterectomy	**Oophorectomy:**<br>3563801, 3563802, 3563803, 3563811, 3563812, 3571307, 3571311, 3571704	**Inclusions:**<br>Hysterectomy: 3565300, 3565301, 3565304, 3565700, 3566100, 3566400, 3566401, 3566700, 3566701, 3567000, 3567302, 3575000, 3575302, 3575600, 3575603, 9044800, 9044801, 9044802<br><br>**Exclusion:**<br>Gynaecological cancers, endometriosis, and risk factors for prophylactic oophorectomy: C56, C796, D27, D391, N801, C541, C539, C55, N802, N851, C772, C570, D069, C578, D069, Z4001, Z4008, N809
Laparoscopic uterine nerve ablation for chronic pelvic pain	3563814	**Inclusions:**<br>Pelvic pain: R102, N731 (females)
Hyperbaric oxygen therapy for various indications	9619100, 1302000, 1302500	**Inclusion:**<br>Acute ankle sprains, carbon monoxide poisoning, Crohn's disease, non-diabetic wounds and ulcers, osteomyelitis, sudden idiopathic hearing loss, peripheral vascular disease, skin grafts and flaps, and cancer: T58, T597, M8617, M8618, M8646, M8666, M8667, M8668, M8687, M8688, M8694, M8695, M8696, M8697, M8698, H903, H904, H905, H912, H919, H931, K50, K500, K501, K508, K509, S934, S9340, S9341, S9342, S9343, K626, L891, L892, L893, L899, L97, L984, S010, S0131, S0188, S211, S3180, S510, S519, S6181, S6188, S619, S810, S817, S8181, S8188, S819, S910, S912, S913, S9181, T013, T793, T813, T8141, T8903, T930, I73, I730, I731, I738, I739, C00 - D48<br>**Exclusion:**<br>Potential sources of radiation injuries, diabetes, osteoradionecrosis, decompression sickness, air or gas embolisms, gas gangrene, and necrotising soft tissue<br>Infections: M8731, M8738, M8785, M8788, M8795, M8798, Z298, K520, K627, L598, L599, M962, N304, T66, Z923, E10-E14, G374, I775, K041, K102, M31, M318, M319, M319, M726, N498, N768, O24, O240, O241, O242, O243, O244, O249, P77, T703, T790, T800, T875, A480, A690

ACHI: Australian Classification of Health Interventions
ICD-10-AM: International Classification of Diseases 10th Revision Australian Modification
Note: We searched for principal and secondary procedures and diagnoses recorded in admissions. Patient 'eligibility' in our study may differ from clinical indication for a procedure.

© 2021 de Oliveira Costa J et al. *JAMA Network Open.*

eTable 2. Number of Public Hospitals Performing Services Measured, and the Number of Low-Value Services Provided Within These Hospitals

	N of hospitals where admitted patients received the service (low-value or not)		Number of low-value services among eligible patients, within hospitals where patients received 5+ services*	
	Any	5+ times	Median	IQR
Knee arthroscopic debridement for osteoarthritis	97	56	3	1 – 7
Vertebroplasty for osteoporotic spinal fractures	29	11	5	2 – 12
Oophorectomy with hysterectomy	75	58	1	0 – 2
Hyperbaric oxygen therapy for various indications	34	11	2	0 – 6
Laparoscopic uterine nerve ablation for chronic pelvic pain	19	3	4	0 - 5

* Public hospitals with at least five admitted patients receiving the service within any episode in their hospital stay between January 2013 to June 2018, irrespective if the service is considered low-value or not

© 2021 de Oliveira Costa J et al. *JAMA Network Open.*

eTable 3. Characteristics of Eligible Inpatients According to Funding Status for Each Measure of Low-Value Service*

Characteristics	Public inpatients<br>n (%)	Private inpatients<br>n (%)
**Knee arthroscopic debridement for osteoarthritis**		
Total number of eligible patients (N)	34,329 (100.0)	4,036 (100.0)
Sex		
Males	13,957 (40.7)	1,504 (37.3)
Females	20,372 (59.3)	2,532 (62.7)
Age group (years old)		
18 - 30	91 (0.3)	≤10 #
31 - 40	203 (0.6)	≤10 #
41 - 50	1071 (3.1)	71 (1.7)
51 - 60	5,152 (15.0)	413 (10.2)
61 - 70	10,811 (31.5)	877 (21.7)
71 - 80	11,274 (32.8)	1,174 (29.1)
80+	5,727 (16.7)	1,489 (36.9)
Remoteness §		
Major Cities	21,035 (61.3)	3,047 (75.5)
Inner Regional	9,244 (26.9)	748 (18.5)
Outer Regional	3,098 (9.0)	185 (4.6)
Remote / Very Remote	147 (0.4)	≤10 #
Interstate / Not defined	805 (2.3)	≤50 #
Socio-economic status (IRSD quintiles) †		
1st quintile (lowest SES)	8,912 (26.0)	≤750 #
2nd quintile	11,584 (33.7)	819 (20.3)
3rd quintile	6,099 (17.8)	812 (20.1)
4th quintile	4,088 (11.9)	672 (16.7)
5th quintile (highest SES)	2,841 (8.3)	935 (23.2)
Interstate / Not defined	805 (2.3)	≤50 #
**Vertebroplasty for osteoporotic spinal fractures**		
Total number of eligible patients (N)	1,591 (100.0)	929 (100.0)
Sex		
Males	395 (24.8)	201 (21.6)
Females	1,196 (75.2)	728 (78.4)
Age group (years old)		
18 - 30	≤10 #	≤10 #
31 - 40	≤10 #	≤10 #
41 - 50	33 (2.1)	≤10 #
51 - 60	112 (7.1)	25 (2.7)
61 - 70	197 (12.4)	75 (8.1)
71 - 80	424 (26.6)	238 (25.6)
80+	812 (51.0)	579 (62.3)
Remoteness §		
Major Cities	1,538 (96.7)	892 (96.0)
Inner Regional	26 (1.6)	26 (2.8)
Outer Regional	≤10 #	≤10 #
Remote / Very Remote	≤10 #	≤10 #
Interstate / Not defined	20 (1.3)	≤10 #
Socio-economic status (IRSD quintiles) †		
1st quintile (lowest SES)	240 (15.1)	≤50 #
2nd quintile	261 (16.4)	98 (10.5)
3rd quintile	421 (26.5)	197 (21.2)
4th quintile	320 (20.1)	176 (18.9)
5th quintile (highest SES)	329 (20.7)	407 (43.8)
Interstate / Not defined	20 (1.3)	≤10 #
**Hyperbaric oxygen therapy for various indications**		
Total number of eligible patients	119,044 (100.0)	43,241 (100.0)
Sex		
Males	59,248 (49.8)	20,989 (48.5)

© 2021 de Oliveira Costa J et al. *JAMA Network Open.*

Females	59,794 (50.2)	22,252 (51.5)
Age group (years old):		
18 - 30	12,830 (10.8)	2,177 (5.0)
31 - 40	11,509 (9.7)	2,830 (6.5)
41 - 50	15,302 (12.9)	4,157 (9.6)
51 - 60	18,138 (15.2)	6,062 (14.0)
61 - 70	19,690 (16.5)	8,565 (19.8)
71 - 80	18,769 (15.8)	8,767 (20.3)
80+	22,806 (19.2)	10,683 (24.7)
Remoteness §		
Major Cities	103,198 (86.7)	38,538 (89.1)
Inner Regional	11,932 (10.0)	3,563 (8.2)
Outer Regional	1662 (1.4)	453 (1.1)
Remote/Very Remote	105 (0.1)	26 (0.1)
Interstate/Not defined	2,147 (1.8)	661 (1.5)
Socio-economic status (IRSD quintiles) †		
1st quintile (lowest SES)	24,283 (20.4)	4,548 (10.5)
2nd quintile	24,870 (20.9)	6,185 (14.3)
3rd quintile	25,672 (21.6)	8,652 (20.0)
4th quintile	20,196 (17.0)	7,916 (18.3)
5th quintile (highest SES)	21,876 (18.4)	15,278 (35.3)
Interstate / Not defined	2147 (1.8)	662 (1.5)
**Oophorectomy with Hysterectomy**		
Total number of eligible patients (N)	14,347 (100.0)	1,569 (100.0)
Females	14,347 (100.0)	1,569 (100.0)
Age group (years old)		
18 - 30	285 (2.0)	14 (0. 9)
31 - 40	2371 (16.5)	215 (13.7)
41 - 50	6476 (45.1)	650 (41.4)
51 - 60	2577 (18.0)	319 (20.3)
61 - 70	1598 (11.1)	213 (13.6)
71 - 80	892 (6.2)	122 (7.8)
80+	148 (1.0)	36 (2.3)
Remoteness §		
Major Cities	8,736 (60.9)	1,105 (70.4)
Inner Regional	3,823 (26.6)	278 (17.7)
Outer Regional	1,373 (9.6)	152 (9.7)
Remote / Very Remote	90 (0.6)	≤10 #
Interstate / Not defined	325 (2.3)	≤30 #
Socio-economic status (IRSD quintiles) †		
1st quintile (lowest SES)	3,837 (26.7)	329 (21.0)
2nd quintile	4,991 (34.8)	349 (22.2)
3rd quintile	2,557 (17.8)	270 (17.2)
4th quintile	1,721 (12.0)	301 (19.2)
5th quintile (highest SES)	916 (6.4)	292 (18.6)
Interstate / Not defined	325 (2.3)	28 (1.8)
**Laparoscopic uterine nerve ablation for chronic pelvic pain**		
Total number of eligible patients (N)	694 (100.0)	82 (100.0)
Females	694 (100.0)	82 (100.0)
Age group (years old)		
18 - 30	296 (42.7)	31 (37.8)
31 - 40	226 (32.6)	23 (28.0)
41 - 50	134 (19.3)	20 (24.4)
51 - 60	25 (3.6)	≤10 #
61 - 70	≤10 #	≤10 #
71 - 80	≤10 #	≤10 #
80+	≤10 #	≤10 #
Remoteness §		
Major Cities	483 (69.6)	42 (51.2)
Inner Regional	99 (14.3)	21 (25.6)
Outer Regional	≤10 #	≤10 #

© 2021 de Oliveira Costa J et al. *JAMA Network Open.*

Remote / Very Remote	≤10 #	≤10 #
Interstate / Not defined	104 (15.0)	14 (17.1)
Socio-economic status (IRSD quintiles) †		
1st quintile (lowest SES)	146 (21.0)	≤10 #
2nd quintile	157 (22.6)	≤10 #
3rd quintile	37 (5.3)	≤10 #
4th quintile	238 (34.3)	39 (47.6)
5th quintile (highest SES)	12 (1.7)	≤10 #
Interstate / Not defined	104 (15.0)	14 (17.1)

* People could have multiple admissions and be categorised in both private and public groups. We present the characteristics of the first admission within the study period for each category patients were eligible.

# For privacy reasons, we used consequential cell suppression for cells with small numbers (≤10) and subsequent cells which would allow for back calculation.

§ Remoteness according to Australian Bureau of Statistics Remoteness Areas of the area (Statistical Area 2) of residence

† SES according to Australian Bureau of Statistics Socio-Economic Indexes for Areas (SEIFA) Index of Relative Socio-Economic Disadvantage (IRSD) of the area (Statistical Area 2) of residence

© 2021 de Oliveira Costa J et al. *JAMA Network Open.*

eFigure. Crude Rates of Low-Value Services (per 1000 Eligible Patients) in Private Compared With Public Inpatients Within Each Public Hospital*

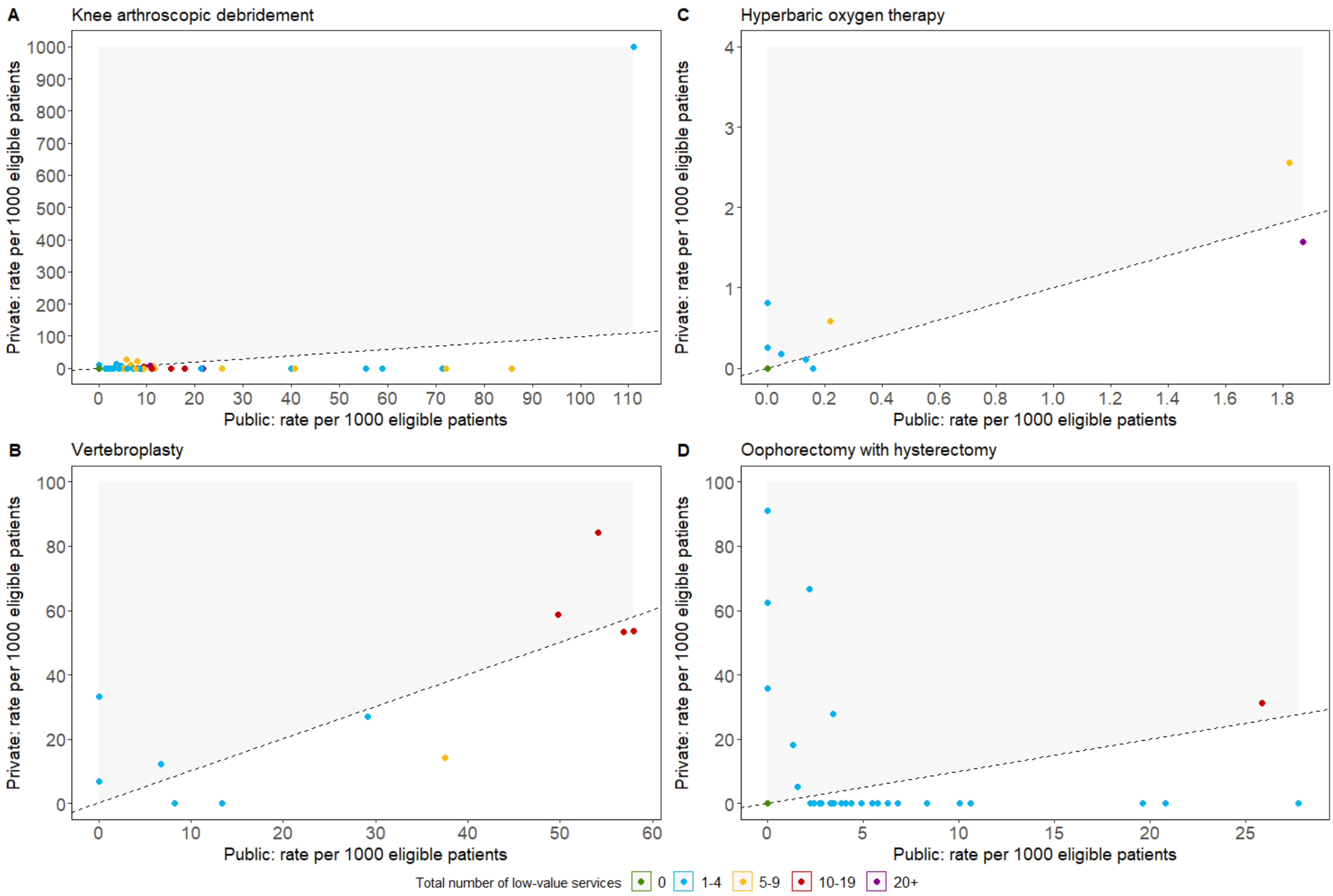

Hospitals in the shaded area represent those with higher rates of low-value services among private than public inpatients.

* Public hospitals with at least five admitted patients receiving the service between January 2013 to June 2018, irrespective if the service is considered low-value or not

© 2021 de Oliveira Costa J et al. *JAMA Network Open.*
